# A Novel Gain-of-Function Nav1.9 Mutation in a Child With Episodic Pain

**DOI:** 10.3389/fnins.2019.00918

**Published:** 2019-09-03

**Authors:** Jianying Huang, Mark Estacion, Peng Zhao, Fadia B. Dib-Hajj, Betsy Schulman, Angela Abicht, Ingo Kurth, Knut Brockmann, Stephen G. Waxman, Sulayman D. Dib-Hajj

**Affiliations:** ^1^Department of Neurology, Center for Neuroscience and Regeneration Research, Yale University School of Medicine, New Haven, CT, United States; ^2^Rehabilitation Research Center, Veterans Affairs Connecticut Healthcare System, West Haven, CT, United States; ^3^Medizinisch Genetisches Zentrum, Munich, Germany; ^4^Department of Neurology, Friedrich-Baur-Institute, Ludwig-Maximilians-University of Munich, Munich, Germany; ^5^Medical Faculty, Institute of Human Genetics, RWTH Aachen University, Aachen, Germany; ^6^Department of Pediatrics and Pediatric Neurology, Georg August University, Göttingen, Germany

**Keywords:** channelopathy, DRG, pain, sodium channel, *SCN11A*, excitability

## Abstract

Voltage-gated sodium channel Nav1.9 is a threshold channel that regulates action potential firing. Nav1.9 is preferentially expressed in myenteric neurons, and small-diameter dorsal root ganglion (DRG) and trigeminal ganglion neurons including nociceptors. Recent studies have demonstrated a monogenic Mendelian link of Nav1.9 to human pain disorders. Gain-of-function variants in Nav1.9, which cause smaller depolarizations of RMP, have been identified in patients with familial episodic pain type 3 (FEPS3) and the more common pain disorder small fiber neuropathy. To explore the phenotypic spectrum of Nav1.9 channelopathy, here we report a new Nav1.9 mutation, N816K, in a child with early-onset episodic pain in both legs, episodic abdominal pain, and chronic constipation. Sequencing of further selected pain genes was normal. N816K alters a residue at the N-terminus of loop 2, proximal to the cytoplasmic terminus of transmembrane segment 6 in domain II. Voltage-clamp recordings demonstrate that Nav1.9-N816K significantly increases current density and hyperpolarizes voltage-dependence of activation by 10 mV, enabling a larger window current. Current-clamp recordings in DRG neurons shows that N816K channels depolarize RMP of small DRG neurons by 7 mV, reduce current threshold of firing an action potential and render DRG neurons hyperexcitable. Taken together these data demonstrate gain-of-function attributes of the newly described N816K mutation at the channel and cellular levels, which are consistent with a pain phenotype in the carrier of this mutation.

## Introduction

Voltage-gated sodium channel Nav1.9 is preferentially expressed in small-diameter sensory neurons of dorsal root ganglia (DRG) and trigeminal ganglia, which include high-threshold nociceptors (Dib-Hajj et al., 1998). Nav1.9 channels can be detected along axons of non-myelinated fibers in sciatic nerve and cornea (Fjell et al., 2000; Black and Waxman, 2002; O’Brien et al., 2008), as well as in myenteric sensory neurons implicating Nav1.9 channel in intestinal reflex function (Rugiero et al., 2003; Coste et al., 2004; Padilla et al., 2007; Osorio et al., 2014). Nav1.9 channels in small DRG neurons from rodents and human produce a tetrodotoxin-resistant (TTX-R) current with distinctive biophysical properties compared to other voltage-gated sodium channels, including hyperpolarized voltage-dependence of activation (at approximately −80 mV), substantial window current (i.e., overlap of activation and steady-stated inactivation) in the voltage domain that includes resting membrane potential (RMP) of DRG neurons, marked persistent current and activation and inactivation slow kinetics (Cummins et al., 1999; Dib-Hajj et al., 1999). The large window current of Nav1.9 channel at physiologically relevant potentials suggests that the channel plays a significant role in regulating neuronal excitability. The anatomical distribution and functional properties support a role for Nav1.9 as a threshold channel (Bennett et al., 2019).

An increasing number of Nav1.9 mutations has been identified in patients with enhanced as well as diminished pain perception. Nav1.9 mutations identified in patients with adult-onset painful neuropathy or early-onset familial episodic pain type 3 (FEPS3) render small DRG neurons hyperexcitable, underlying enhanced pain as experienced by the patients (Zhang et al., 2013; Huang et al., 2014; Han et al., 2015, 2017; Leipold et al., 2015; Okuda et al., 2016). Gain-of-function attributes of these Nav1.9 mutants include a hyperpolarizing shift in voltage-dependence of activation by 6–10 mV, compared to wild-type channels (Huang et al., 2014; Han et al., 2015, 2017). An intriguing finding is that the gain-of-function mutations in transmembrane segment 6 of domain I (DI/S6-L396P), DII/S6-L811P, and DIII/S6-L1302F in Nav1.9, which massively hyperpolarize activation by approximately 22–27 mV, are found in subjects with a loss-of-pain perception (Leipold et al., 2013; Huang et al., 2017; King et al., 2017). The resulting increase of window current from such a massive hyperpolarizing shift in activation deeply depolarizes RMP of DRG neurons, leading to neuronal inexcitability at the cellular level, which underlies painless injuries at the clinical level (Huang et al., 2017). Thus, a spectrum of clinical phenotypes is produced by gain-of-function mutations in Nav1.9.

Here we report a novel mutation in human Nav1.9 channel found in a child with FEPS3. The N816K mutation is located at the juncture between transmembrane segment 6 of domain II (DII/S6) and L2, the loop that joins domains II and III, in proximity to the hydrophobic ring that stabilizes the closed state of sodium channels, which is formed by aromatic residues at the C terminus of each of the S6 segments in the four domains (Lampert et al., 2008). We characterized changes in biophysical properties of the mutant channel using voltage-clamp recordings and assessed firing properties of DRG neurons using current-clamp recordings. This study expands the spectrum of Nav1.9 channelopathies in patients with pain.

## Materials and Methods

### Plasmid

The human wild-type GFP-2A-Na_v_1.9 plasmid was previously described (Huang et al., 2014). Briefly, a sequence encoding enhanced green fluorescent protein (EGFP) was cloned upstream of the human Na_v_1.9 ATG with a “stopGo” 33 amino acid 2A linker, which allows the GFP-2A adaptor and the Na_v_1.9 channel proteins to be produced as independent proteins from the same mRNA (Ryan and Drew, 1994; Atkins et al., 2007; Luke et al., 2008). The c.2448T > A (p.N816K) was introduced into the construct using QuikChange^®^ II XL site-directed mutagenesis (Stratagene, La Jolla, CA, United States) and referred to as N816K channels hereinafter.

### Primary Sensory Neuron Isolation and Transfection for Whole-Cell Patch-Clamp Recordings

Animal studies followed a protocol approved by Department of Veterans Affairs West Haven Medical Center Animal Use Committees. For voltage-clamp recording, DRG neurons were isolated from homozygous Nav1.9^–/–^ mice (4–8 weeks old, both male and female) that lack endogenous Nav1.9 and transfected by electroporation as previously reported (Dib-Hajj et al., 2009). Briefly, DRGs were rapidly harvested, incubated at 37°C for 20 min in complete saline solution (CSS) [in mM: 137 NaCl, 5.3 KCl, 1 MgCl2, 25 sorbitol, 3 CaCl2, and 10 N-2-hydroxyethylpiperazine-N′-2-ethanesulfonic acid (Hepes), adjusted to pH 7.2 with NaOH] containing 0.5 U/mL Liberase TM (Sigma) and 0.6 mM EDTA, followed by a 15 min incubation at 37°C in CSS containing 0.5 U/mL Liberase TL (Sigma), 0.6 mM EDTA, and 30 U/mL papain (Worthington Biochemical). DRGs were then centrifuged and triturated in 0.5 mL of DRG media: Dulbecco’s modified Eagle’s medium-F12 (1:1) with 100 U/mL penicillin, 0.1 mg/mL streptomycin (Invitrogen), and 10% fetal bovine serum (Hyclone), containing 1.5 mg/mL BSA (low endotoxin; Sigma) and 1.5 mg/mL trypsin inhibitor (Sigma). After trituration, the cells were transfected with WT or mutant Nav1.9 constructs using a Nucleofector IIS (Lonza) and Amaxa Basic Neuron SCN Nucleofector Kit (VSPI-1003), as described before (Huang et al., 2014, 2017). Briefly, the cell suspension was centrifuged (100 × g for 3 min), and the cell pellet was resuspended in 20 μL of Nucleofector solution, mixed with 2.5 μg of WT or N816K human Nav1.9 channels, and transfected using Nucleofector IIS and protocol SCN-BNP 6. After electroporation, 100 μL of calcium-free DMEM (37°C) was added, and cells were incubated at 37°C for 5 min to recover. The cell mixture was then diluted with DRG media containing 1.5 mg/mL BSA (low endotoxin) and 1.5 mg/mL trypsin inhibitor (Sigma), seeded onto poly-D-lysine/laminin-coated coverslips (BD), and incubated at 37°C in a 95% air/5% CO2 (vol/vol) incubator for 45 min for neurons to attach to the coverslips. After 45 min, 0.9 mL of DRG media supplemented with nerve growth factor (50 ng/mL) and glial cell line-derived neurotrophic factor (50 ng/mL) was added into each well, and the DRG neurons were maintained at 37°C in a 95% air/5% CO_2_ (vol/vol) incubator. Voltage-clamp recordings were performed approximately 65–75 h post-transfection.

For current-clamp recording, DRGs from 4 to 6 weeks old female and male Sprague-Dawley rats were harvested and dissociated as described previously (Dib-Hajj et al., 2009). Briefly, DRG neurons were dissociated with a 20 min incubation in 1.5 mg/mL Collagenase A (Roche) and 0.6 mM EDTA, followed by a 17 min incubation in 1.5 mg/mL Collagenase D (Roche), 0.6 mM EDTA, and 30 U/mL papain; DRGs were then centrifuged and triturated in 0.5 mL of DRG media containing 1.5 mg/mL BSA (low endotoxin) and 1.5 mg/mL trypsin inhibitor (Sigma). After trituration, 2 mL of DRG media was added to the cell suspension, which was filtered with 70 μm nylon mesh cell strainer (Becton Dickinson). The mesh was washed twice with 2 mL of DRG media. The cells were then transfected as described above for voltage-clamp recording with 2.0 μg of WT or N816K hNav1.9 channels. After transfection, cells were recovered in calcium-free DMEM, fed with DRG media supplemented with nerve growth factor (50 ng/mL) and glial cell line-derived neurotrophic factor (50 ng/mL), and maintained at 37°C in a 95% air/5% CO_2_ (vol/vol) incubator for 45–55 h before current-clamp recording.

### Whole-Cell Patch-Clamp Recordings

Voltage-clamp recordings were obtained at 22 ± 1°C using an EPC-10 amplifier (HEKA Electronics, Lambrecht/Pfalz, Germany). Fire-polished electrodes were fabricated from 1.6 mm outer diameter borosilicate glass micropipettes (World Precision Instruments) using a Sutter Instruments P-97 puller and had a resistance of 0.8–1.3 MΩ. Transfected small DRG neurons (<25 μm) from Nav1.9^–/–^ mice with robust green fluorescence and no apparent neurites were selected for voltage-clamp recording. The average diameter for small DRG neurons overexpressing wild-type hNav1.9 channels is not significantly different from those overexpressing N816K mutant hNav1.9 channels (WT: 21.0 ± 0.68 μm, *n* = 18; N816K: 21.2 ± 0.65 μm, *n* = 20; *p* = 0.804). The extracellular bath solution contained the following (in mM): 140 NaCl, 3 KCl, 1 MgCl_2_, 1 CaCl_2_, 10 HEPES, 5 CsCl, 20 tetraethylammonium chloride (TEA⋅Cl), pH 7.3 with NaOH. Osmolarity was adjusted to 320 mOsmol/L with sucrose. Tetrodotoxin (1 μM), CdCl_2_ (0.1 mM), and 4-aminopyridine (1 mM) were added to block endogenous tetrodotoxin-sensitive sodium currents, calcium currents, and potassium currents, respectively. The intracellular pipette solution contained the following (in mM): 140 CsF, 10 NaCl, 1 EGTA, 10 HEPES, and 10 dextrose, pH 7.3 with CsOH. Osmolarity was adjusted to 310 mOsmol/L with sucrose. Sodium currents were acquired with Patchmaster at 5 min after establishing whole-cell configuration, sampled at 50 kHz, and filtered at 2.9 kHz.

Under whole-cell voltage-clamp configuration and guided by green fluorescence as evidence for expression of hNav1.9 channels, each individual neuron was held at −120 mV initially to record the total TTX-R sodium currents by a series of 100 ms step voltage commands from −120 to +40 mV in 5 mV increments at 5 s intervals in order to elicit the total TTX-R sodium currents, which consist of hNav1.9 currents and endogenous mouse Nav1.8 TTX-R currents. The same cell was subsequently held at −60 mV to inactivate Nav1.9 channels (Cummins et al., 1999), prepulsed to −120 mV for 100 ms to allow Nav1.8 channels to recover from fast-inactivation, followed by 100 ms step voltage commands from −120 to +40 mV, which elicited the intact endogenous mouse Nav1.8 currents. Nav1.9 currents were generated by subtracting Nav1.8 currents from total TTX-R sodium currents. Linear leak currents were subtracted out using the P/N method. Seventy five to eighty five percent compensation was applied. Cells were excluded from analysis if the predicted voltage error (*Verr*) is greater than 5 mV, calculated by *Verr* = *Ipeak^∗^Rs^∗^(1-Comp%)*, where *Ipeak* is the maximal peak sodium current, *Rs* is the series resistance, and *Comp%* is the percentage of compensation. There is no significant difference in voltage error between WT and N816K (WT: 1.83 ± 0.25 mV, *n* = 13; N816K: 2.37 ± 0.36 mV, *n* = 12; *p* = 0.231). Voltage-dependence of activation for Nav1.9 channels were fit with Boltzmann functions in the form of *G* = *G_max_/(1* + *exp[(V_1__/__2, act_ -V)/k])*, where *G*_max_ is the maximal conductance, *V_1__/__2__,_*_act_ is the voltage at which activation is half-maximal, *V* is the test voltage and *k* is the slope factor. Steady-state fast inactivation was assessed by measuring the remaining non-inactivated channels by depolarizing the cell to -45 mV for 50 ms, a voltage near which peak Nav1.9 currents are recorded, following a series of 500 ms prepulses from -130 to 10 mV in 10 mV increments. Normalized peak inward currents were fit with Boltzman functions: *I/I_max_* = *A* + (1-*A*)/(1 + *exp*[(*V -V*_1__/__2_*_, fast_*)/k]), where *V* represents the inactivating prepulse voltage, *V*_1__/__2__, fast_ represents the midpoint of the steady-state fast inactivation.

Action potentials were recorded by current-clamp experiments in adult rat small (<30 μm in diameter) DRG neurons transfected with WT or N816K mutant hNav1.9 channels, guided by green fluorescence. The average diameter for neurons expressing WT or N816K is 27.5 ± 0.27 (*n* = 39), and 27.0 ± 0.25 (*n* = 43) (*p* = 0.155), respectively. Electrodes had a resistance of 1.0–1.8 MΩ when filled with the pipette solution, which contained the following (in mM): 140 KCl, 0.5 EGTA, 5 HEPES, 10 dextrose, and 3 Mg-ATP, pH 7.3 with KOH. Osmolarity was adjusted to 310 mOsm/L with sucrose. The extracellular solution contained the following (in mM): 140 NaCl, 3 KCl, 2 MgCl_2_, 2 CaCl_2_, 10 HEPES, and 10 dextrose, pH 7.3 with NaOH. Osmolarity was adjusted to 320 mOsm/L with sucrose. Whole-cell configuration was obtained in voltage-clamp mode before proceeding to the current-clamp recording mode. Threshold was determined by the first action potential elicited by a series of 200 ms depolarizing current injections that increased in 5 pA increments. Repetitive firing was evaluated by a series of 500 ms depolarizing current injections from 25 to 500 pA in 25 pA increments. Neurons that fired spontaneously (i.e., without external current stimuli) were excluded from analysis of RMP, input resistance, current threshold, amplitude, half-width, or after-hyperpolarization potential.

### Data Analysis

Electrophysiological data were analyzed using Fitmaster (HEKA Electronics) and Origin 9 (Microcal, Northampton, MA, United States), and presented as means ± standard error. Statistical significance was determined by independent Student’s *t*-tests. Mann–Whitney test was used to compare frequency of evoked firing in response to graded external stimuli. Two-proportion *z*-test was used in comparisons of proportion of cells producing spontaneous activity and proportion of cells firing repetitively). A *p-*value of less than 0.05 is considered statistically significant.

## Results

### Clinical Phenotype

Informed consent of the parents of this child was obtained before the initiation of the study, which was approved by the local ethics committee at the Uniklinik under an approved institutional review board. The novel N816K mutation in human Nav1.9 channel was found in a 7 years old girl with familial episodic pain in her legs with onset at 6 months of age. At the time of study she complained of massive pain that occurred every evening; she reported that naproxen (NSAID) alleviated the pain. In addition, she described episodes of abdominal pain with onset at 6 years of age, and a history of chronic constipation since her first months of life. Diagnostic work-up including rectal biopsy revealed no abnormal findings. Nerve conduction studies were normal. Sweating was normal, and there was no sensory disturbance. The clinical features of this girl corresponded to the descriptions of familial episodic pain type 3 (FEPS3; OMIM #615552) associated with heterozygous *SCN11A* variants.

Motor development of this girl was mildly slower than in her older sister; she walked without support at 18 months of age. At age 7 years, muscle strength and motor endurance were reduced. Her maximal walking distance was 200 m during daytime and 100 m in the evening, after which she then needed to sit down for a short break. Cold or heat did not trigger pain episodes or influence motor performance. On physical examination, there were mild muscular hypotonus, joint hypermobility and mild proximally pronounced muscle weakness. Muscle stretch reflexes were normal.

A molecular genetic panel for painful neuropathy showed the heterozygous variant c.2448T > A; p.(Asn816Lys); N816K in the *SCN11A* gene (ENST00000302328). This extremely rare variant (rs376128467) has been reported only once in 250,714 alleles in the Genome Aggregation database (gnomAD)^1^, and has not been reported among the few patients with episodic pain due to *SCN11A* mutations. Further genetic testing included *SCN9A*, *SCN10A*, *TRPA1* – all with normal results. Interestingly, the father of the proband is a carrier of this mutation but has not experienced any of these symptoms.

### Biophysical Properties of N816K Mutant hNav1.9 Channel

**FIGURE 1:**
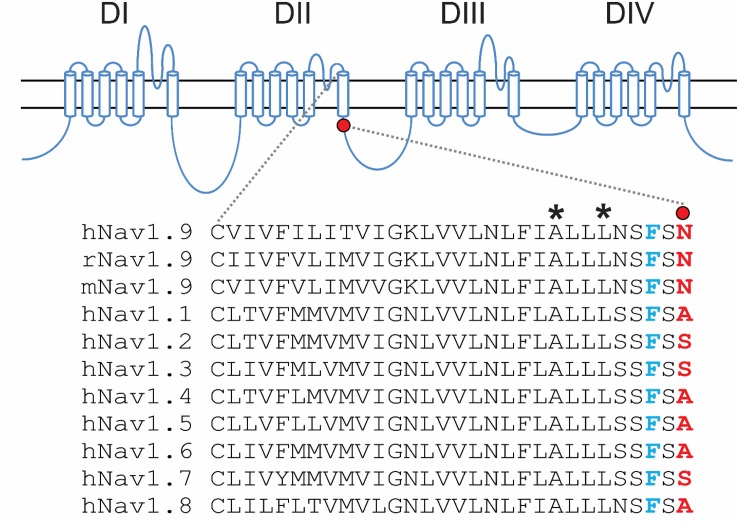
Schematic illustration of human voltage-gated sodium channels and location of the N816K mutation. A functional Nav channel consists of four domains (DI-DIV) and each domain consists of six transmembrane segments (S1–S6). Intracellular loop L2 connects DII to DIII. Sequence alignment of DII/S6 and beginning of L2 from all human Navs shows the location of the N816K mutation. N816K mutation (red) is separated by one residue from the gating phenylalanine residue (blue), which contributes to the hydrophobic ring at the cytoplasmic aperture of Nav channels, stabilizing the closed state of the channel (Lampert et al., 2008). Previous studies reported two gain-of-function mutations, A808G (Zhang et al., 2013) and L811P (Leipold et al., 2013), in hNav1.9 channels residing in DII/S6, which are marked with asterisks.

The N816K substitution alters a residue at the N-terminus of loop 2, proximal to the cytoplasmic terminus of transmembrane segment 6 in domain II (DII/S6) (Figure 1). While N816 is conserved in Nav1.9 rodent orthologs, it is not conserved in the other human voltage-gated sodium channels, where the corresponding residue is either an alanine or a serine (Figure 1). The location of N816 is one residue away from the highly conserved phenylalanine that has been implicated in forming the hydrophobic ring with the corresponding residues in S6 segments of the other domains, and which stabilizes the closed state of the channel (Lampert et al., 2008).

**FIGURE 2:**
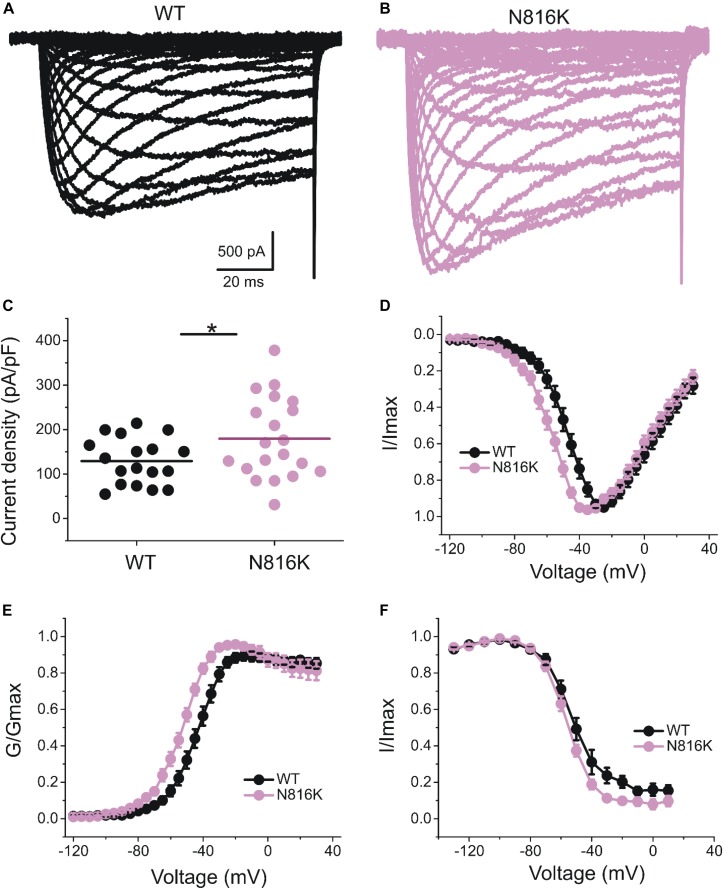
N816K mutation hyperpolarizes voltage-dependence of activation of hNav1.9 channels in small DRG neurons. Representative traces showing characteristic human Nav1.9 currents from small DRG neurons expressing **(A)** wild-type (WT) or **(B)** N816K mutant Nav1.9 channels in adult Nav1.9-null mice. **(C)** Scatter plot of current densities, determined by normalization of peak current to cell capacitance, in small DRG neurons expressing WT and N816K mutant hNav1.9 channels. Solid lines indicate mean current densities. ^∗^*p* < 0.05. **(D)** Current-voltage relationships (I–V curves) for WT and N816K mutant hNav1.9 channels. Normalized peak inward current was plotted against voltage commands. N816K mutation hyperpolarizes the I–V curve of Nav1.9 channels. Boltzmann fits for activation **(E)** and steady-state fast-inactivation **(F)** show that N816K hyperpolarizes voltage-dependence of activation of Nav1.9 channels, but does not significantly change voltage-dependence of steady-state fast inactivation.

The introduction of a positive charge proximal to the hydrophobic ring suggested an impact on channel gating. We investigated the effect of N816K mutation on biophysical properties of human Nav1.9 channels (hNav1.9 hereafter) when expressed in small DRG neurons from adult Nav1.9^–/–^ mice. Representative persistent TTX-R current traces for wild-type (WT) and N816K hNav1.9 channels are shown in Figures 2A,B, respectively. Figure 2C shows that N816K mutation lead to a significant increase in current density by 39% (WT: −129 ± 12 pA/pF, *n* = 18; N816K: −179 ± 20 pA/pF, *n* = 20; *p* = 0.0467) (Table 1). The current-voltage (I–V) relationship of Nav1.9 channels is shown in Figure 2D. Normalized peak currents of WT and N816K channels were plotted against the voltage. Voltage-dependence of activation for Nav1.9 channels was shifted in a hyperpolarizing direction by the N816K mutation (Figure 2E). The activation midpoint of N816K mutant channel was significantly hyperpolarized by approximately 10 mV (WT: −44.7 ± 2.2 mV, *n* = 13; N816K: −54.6 ± 1.6 mV, *n* = 12; *p* = 0.00164), but the slope factor was unaffected (WT: 7.21 ± 0.64 mV, *n* = 13; N816K: 7.87 ± 0.46 mV, *n* = 12; *p* = 0.418) (Table 1). There was no statistical significance in midpoint voltage (WT: −52.4 ± 3.3 mV, *n* = 10; N816K: −56.1 ± 1.5 mV, *n* = 10; *p* = 0.321) or the slope factor (WT: 7.41 ± 0.37 mV, *n* = 10; N816K: 7.66 ± 0.41 mV, *n* = 10; *p* = 0.656) for steady-state fast-inactivation between WT and N816K mutant channels (Table 1). The fraction of non-inactivating channel for N816K mutant channel was reduced but it did not reach statistical significance (WT: 15.2 ± 3.0, *n* = 10; N816K: 8.56 ± 2.1, *n* = 10; *p* = 0.0857) (Figure 2F).

**TABLE 1:** Biophysical properties of wild-type (WT) and mutant Na_v_1.9 channels in small DRG neurons.

**Nav1.9**	**Current density**		**Activation (mV)**			**Steady-state fast inactivation (mV)**			
			
	**pA/pF**	**n**	**V_1__/__2__, act_**	**k**	**n**	**V_1__/__2__,fast_**	**k**	**A%**	**n**
WT	−129 ± 12	18	−44.7 ± 2.2	7.21 ± 0.64	13	−52.4 ± 3.3	7.41 ± 0.37	15.2 ± 3.0	10
N816K	−179 ± 20^∗^	20	−54.6 ± 1.6^∗∗^	7.87 ± 0.46	12	−56.1 ± 1.5	7.66 ± 0.41	8.56 ± 2.1	10
*p*-value	0.0467		0.00164	0.418		0.321	0.656	0.0857	

**^∗^p < 0.05; ^∗∗^p < 0.01 versus WT channels.**

### DRG Neuronal Excitability Is Increased by N816K Mutant Channels

**FIGURE 3:**
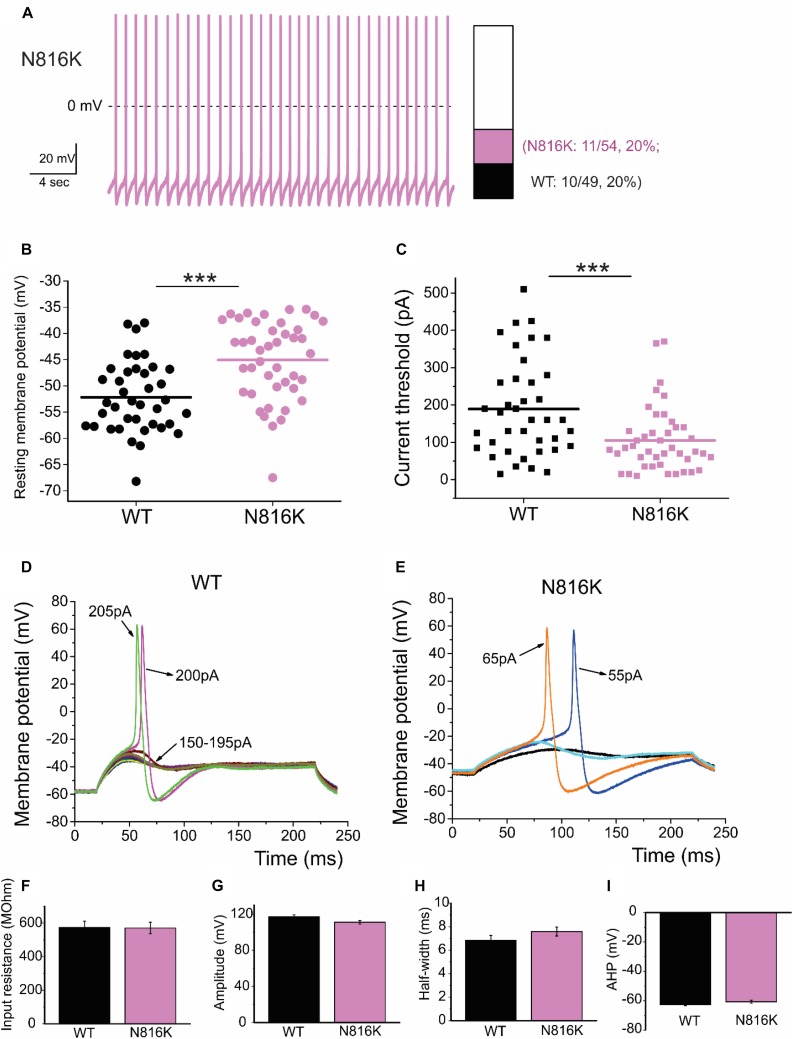
N816K mutation depolarizes resting membrane and reduces threshold for action potentials in small DRG neurons. **(A)** A representative 30 s trace from a cell expressing N816K mutant channels demonstrates spontaneous firing of action potentials (left panel). However, N816K mutant channels do not change the proportion of spontaneously-firing cells, compared to WT hNav1.9 channels (right panel). **(B)** Scatter plot of resting membrane potential (RMP) in small DRG neurons expressing WT or N816K mutant hNav1.9 channels. Solid lines indicate mean RMP. ^∗∗∗^*p* < 0.001. **(C)** Scatter plot of current threshold, the minimal current injection required to elicit an action potential in small DRG neurons expressing WT or N816K mutant hNav1.9 channels. Solid lines indicate mean RMP. ^∗∗∗^*p* < 0.001. **(D)** Representative action potential traces recorded from a small DRG neuron expressing WT hNav1.9 channels show that the cell produces subthreshold depolarizations in response to current stimuli from 150 to 195 pA, and does not generate an all-or-none action potential until the injected current reaches 200 pA, which is defined as its current threshold. **(E)** Representative action potential traces recorded from a small DRG neuron expressing N816K mutant hNav1.9 channels show that the current threshold for this cell is 55 pA. **(F)** There is no significant difference in input resistance between WT and N816K mutant hNav1.9 channels. N816K mutation does not alter the waveforms of action potentials, indicated by **(G)** action potential amplitude, **(H)** after-hyperpolarization potential, or **(I)** half-width of action potentials.

**FIGURE 4:**
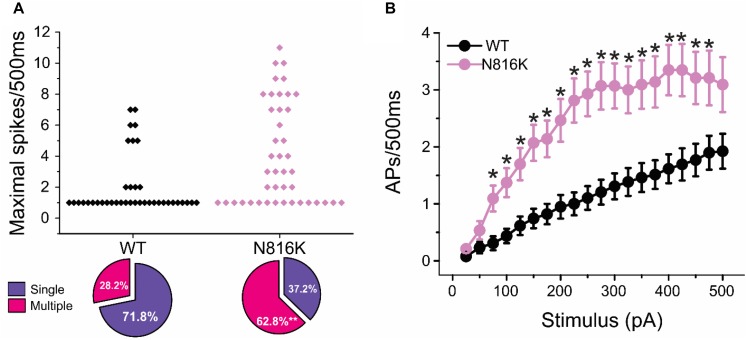
N816K mutation enables hyperexcitability in small DRG neurons. **(A)** Scatter plot of maximal numbers of action potentials evoked by stimuli ranging from 25 to 500 pA in cells expressing WT and N816K mutant hNav1.9 channels. Cells are divided into two groups, those that only fire a single action potential during 500 ms course and those that fire multiple action potentials. N816K mutation increases the proportion of cells that fire multiple action potentials, compared to WT hNav1.9 channels. **(B)** Average action potential frequency during 500 ms current injections ranging from 25 to 500 pA in 25 pA increments. ^∗^*p* < 0.05.

**FIGURE 5:**
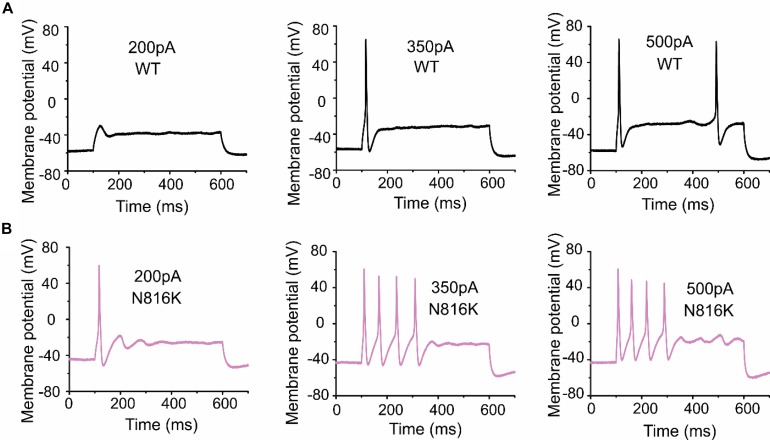
A small DRG neuron expressing N816K mutant hNav1.9 channels produces more action potentials than WT in response to the same stimuli. Representative action potential traces from a small DRG neuron expressing **(A)** WT or **(B)** N816K mutant hNav1.9 channels in response to 200, 350, and 500 pA current stimuli, respectively.

To assess the effects on the firing properties of DRG neurons resulting from the changes in biophysical properties of Nav1.9-N816K channels, we studied excitability of small DRG neurons expressing either WT or N816K mutant hNav1.9 channel using current-clamp recordings. Figure 3A (left) shows a representative trace of spontaneous firing of action potentials, in the absence of external current stimulus, in a small DRG neuron expressing N816K mutant channel. Expression of N816K mutant channels did not change the percentage of spontaneously-firing neurons (WT: 10 out of 49 cells, 20%; N816K, 11 out of 54 cells, 20%, *p* > 0.999) (Figure 3A, right). RMP was significantly depolarized by 7 mV in small DRG neurons expressing N816K as compared to WT channel (WT: −52.2 ± 1.1 mV, *n* = 39; N816K: -45.1 ± 1.2 mV, *n* = 43; *p* < 0.001) (Table 2 and Figure 3B). Expression of N816K channels also significantly reduced current threshold of action potential firing by 44% (WT: 189 ± 21 pA, *n* = 39; N816K: 105 ± 13 pA, *n* = 43; *p* < 0.001) (Table 2 and Figure 3C). Figures 3D,E show representative action potential traces in neurons expressing WT and N816K mutant channels, respectively. The DRG neuron expressing WT Nav1.9 channel in Figure 3D did not fire until the current stimulus reached 200 pA, which was defined as its current threshold, while expression of N816K channels lowered the current threshold to 55 pA as shown in Figure 3E. In contrast to RMP depolarization and current threshold reduction by N816K mutant channel, there was no significant difference in input resistance (WT: 574 ± 37 MΩ, *n* = 39; N816K: 571 ± 34 MΩ, *n* = 43; *p* = 0.945) (Figure 3F), amplitude of action potential (WT: 117 ± 2.3 mV, *n* = 39; N816K: 111 ± 2.0 mV, *n* = 43; *p* = 0.0650) (Figure 3G), half-with of action potentials (WT: 6.84 ± 0.41 ms, *n* = 39; N816K: 7.59 ± 0.38 mV, *n* = 43; *p* = 0.183) (Figure 3H), or after-hyperpolarization potential (AHP) (WT: -62.6 ± 0.77 mV, *n* = 39; N816K: −60.7 ± 1.0 mV, *n* = 43; *p* = 0.135) (Figure 3I and Table 2). When a series of stimuli of 500 ms ranging from 25 to 500 pA was applied, a significantly greater population of small DRG neurons expressing N816K mutant channels fired multiple action potentials compared to DRG neurons expressing WT channels (WT: 11 out of 39 cells, 28.2%; N816K, 27 out of 43 cells, 62.8%, *p* = 0.0017) (Figure 4A). DRG neurons expressing N816K mutant channel are hyperexcitable as compared to those expressing WT Nav1.9 channels (Figure 4B). Representative traces in Figure 5 show that in response to 200 pA current injection, a cell expressing WT Nav1.9 channel does not fire any all-or-none action potential within 500 ms (Figure 5A) while the other cell expressing N816K mutant channel does (Figure 5B). In response to greater stimuli at 350 and 500 pA, the cell expressing N816K is able to fire more action potentials (Figure 5B) than the one expressing WT Nav1.9 channel (Figure 5A).

**TABLE 2:** Action potential characterization for wild-type (WT) and mutant Na_v_1.9 channels in small DRG neurons.

			**Input**	**Current**			
			**resistance**	**threshold**	**Amplitude**	**Half-width**	
	**Nav1.9**	**RMP (mV)**	**(MΩ)**	**(pA)**	**(mV)**	**(ms)**	**AHP (mV)**
WT	*n* = 39	−52.2 ± 1.1	574 ± 37	189 ± 21	117 ± 2.3	6.84 ± 0.41	−62.6 ± 0.77
N816K	*n* = 43	−45.1 ± 1.2^∗∗∗^	571 ± 34	105 ± 13^∗∗∗^	111 ± 2.0	7.59 ± 0.38	−60.7 ± 1.0
*p*-value		2.58e−5	0.945	7.70e−4	0.0650	0.183	0.135

**^∗∗∗^p < 0.001 versus WT channels.**

## Discussion

We report here a novel mutation on *SCN11A* gene that encodes Nav1.9 channel, identified in a child with FEPS3. Our voltage-clamp experiments demonstrated that the N816K mutation hyperpolarizes the voltage-dependence of activation of Nav1.9 channel by approximately 10 mV, a gain-of-function attribute underlying the proexcitatory effects of this mutation in small DRG neurons. To confirm this, we expressed N816K mutant channel in small DRG neurons and examined their excitability. We found that N816K mutant channels depolarized RMP by 7 mV, reduced current threshold for firing an all-or-none action potential by 44%, and endowed DRG neurons with hyperexcitability in response to external stimuli as compared to the wild-type Nav1.9 channel. Thus, gain-of-function attributes at the channel and cellular levels could explain the pain phenotype in this patient.

Patients with Nav1.9-related pain syndromes or painless injuries have reported GI disturbances in addition to their pain syndrome (Bennett et al., 2019). Na_V_1.9 expression has also been shown in myenteric and submucosal plexuses in normal human colon, and reduced Na_V_1.9 expression has been reported in ganglionic tissue from patients with Hirschsprung’s disease (O’Donnell et al., 2016), supporting a contribution of Na_V_1.9 in GI function. This role of Nav1.9 is consistent with data from animal studies showing a role of this channel in regulating intestinal contraction (Hockley et al., 2014). Our patient reported chronic constipation from an early age, and abdominal pain, and thus conform to the FEPS3 phenotype.

It is remarkable that the proband’s asymptomatic father is a carrier of the N816K mutation. An explanation based on sexual dimorphism is not supported by the reporting of pain in males with FEPS3 (Zhang et al., 2013; Okuda et al., 2016; Han et al., 2017). Variable penetrance is reported for several sodium channel mutations, especially for those with gain-of-function properties (Choi et al., 2010; Estacion et al., 2011). The reason for this high variability remains elusive, but may be explained by modifying genetic factors and/or exogenous triggers such as e.g., diabetes mellitus, infections, neurotoxic agents. However, we did not identify any potential triggers in the index patient nor additional rare variants in Na_V_1.7, Na_V_1.8, or Na_V_1.9 which could have been considered as triggers. This clearly underlines the need for functional characterization of suspicious variants as done in this study to classify them as pathogenic (Waxman et al., 2014).

It is conceivable that the proband carries additional aggravating genetic variants in genes other than sodium channels, or, that the father is a carrier of a protective modifying variant(s) in other genes that mask the effect of the mutation. We have recently shown that a gain-of-function variant in Kv7.2 voltage-gated potassium channel contributes to amelioration of pain in a mother carrying the Nav1.7-S241T mutation which causes inherited erythromelalgia, compared to her son with the same Nav1.7 mutation but wild-type Kv7.2 (Mis et al., 2019). We also showed using dynamic clamp that substituting the M-current produced by the heterotetramer Kv7.2/Kv7.3 attenuates the excitability of sensory neurons that were differentiated from induced pluripotent stem cells (iPSC) of the son, further supporting the view that the gain-of-function mutation in Kv7.2 is protective against severe pain. Current iPSC differentiation strategies have not yielded detectable Nav1.9 currents (Eberhardt et al., 2015); thus studies of the impact of the mutation on the cellular behavior of sensory neurons that are differentiated from patient-specific iPSCs, and to possibly identify pain resilience genes in the father await improvements in differentiation protocols of sensory neurons that lead to the expression of a comparable set of ion channels as in mature sensory neurons.

Motor development in the proband was mildly slower than her older sister. Nav1.9 has been previously reported to be essential for activity-dependent axonal growth of mouse motoneurons in culture (Subramanian et al., 2012). Consistent with this finding, a motor deficit, muscular hypotonia, has been previously reported in patients carrying the L811P gain-of-function mutation in Nav1.9 which is associated with insensitivity to pain (Leipold et al., 2013; Woods et al., 2015; King et al., 2017). However, muscular hypotonia has not been reported in patients carrying gain-of-function mutation in Nav1.9 associated with increased pain (Okuda et al., 2016). Thus, whether the motor developmental delay in this patient with the Nav1.9-N816K gain-of-function mutation is coincidental or causative cannot be ascertained at this time.

Naproxen alleviated the pain for our patient carrying the N816K Nav1.9 mutation, similar to previously identified patients of FEPS3 who were also responsive to anti-inflammatory agents (Zhang et al., 2013; Leipold et al., 2015; Okuda et al., 2016). This suggests an inflammatory component of FEPS3, consistent with a role of Nav1.9 in inflammatory pain. Current density of Nav1.9 channels in DRG neurons was significantly increased upon exposure to PGE2 (Rush and Waxman, 2004), and underwent an approximately sixfold increase after exposure to an inflammatory cocktail that contains bradykinin, ATP, histamine, prostaglandin-E2, and norepinephrine (Maingret et al., 2008). Nav1.9 has been shown to potentiate the effects of oxidized phospholipids on excitability of nociceptors (Martin et al., 2018). Moreover, the engagement of Nav1.9 channel is required for developing inflammatory hypersensitivity. Thermal but not mechanical hypersensitivity was substantially diminished in Nav1.9^–/–^ mice under peripheral inflammatory condition (Amaya et al., 2006). Heat and mechanical hypersensitivity were compromised in mice knocked down of Nav1.9 following subacute paw inflammation and chronic ankle inflammation (Lolignier et al., 2011). Excitatory responses to inflammatory mediators and subsequent mechanical hypersensitivity in colonic afferents have been reported to be attenuated in Nav1.9^–/–^ mice (Hockley et al., 2014), and the hypersensitivity of pelvic afferents in response to prostaglandin E2 was also diminished in Nav1.9^–/–^ mice (Ritter et al., 2009). These data from human and animal studies confirm a role for Nav1.9 in inflammatory pain, and suggest that data from preclinical animal studies might be translatable to humans.

Previous studies have characterized gain-of-function mutations A808G and L811P, which are located a few amino acid residues upstream of N816K. When expressed in ND7/23 cells, L811P shifted the voltage-dependence of activation by −28 mV and significantly increased the current density (Leipold et al., 2013). The A808G mutation increased current density of the channel without altering activation, however, voltage-dependence of activation was assessed from −90 to −50 mV (Zhang et al., 2013); interpretation of the effect on voltage dependence is limited, however, because Nav1.9 activation does not peak at −50 mV as shown in our current study (Figure 2E). Nonetheless, N816K, along with A808G and L811P, within DII/S6 or near the juncture with L2, all demonstrate gain-of-function attributes in Nav1.9 channel, implicating structural significance of this region of interest in channel gating.

The mutation replaces an asparagine residue (N816) located in L2 proximal to the cytoplasmic terminus of DII S6 with a positively charged residue lysine (K816). The functional impact of this substitution cannot be simply inferred from sequence analysis because of the divergence of the corresponding residue among other human voltage-gated sodium channels (Ala or Ser residues, Figure 1). However, N816 is close to a highly conserved phenylalanine F814, which corresponds to F960 in hNav1.7, which participates in forming the hydrophobic ring lining the cytoplasmic aperture of Nav1.7, along with corresponding residues in the S6 segments of the other domains (Lampert et al., 2008), including the inherited erythromelalgia mutation DIV/F1449 (Dib-Hajj et al., 2005). Destabilization of the hydrophobic ring by the F1449V or F960V causes hyperpolarized activation of the mutant Nav1.7 channels, which we interpreted as a destabilization of the closed-state of the channel (Lampert et al., 2008). Nav1.9/F814 is only two amino acids away (∼10 Å) and the aromatic ring moiety will likely sense the nearby charge and form a dipole that would not occur with the normal N816 residue. This increased interaction induced by the N816K mutation could destabilize the closed state and contribute to the hyperpolarizing shift of activation voltage-dependence of the mutant channel, compared to wild-type Nav1.9. Alternatively, the interaction of the mutant channel with the membrane phospholipid bilayer could be altered by the introduction of the lysine residue at the cytoplasmic phase of the mutant channel pore. It has been previously shown that the interaction between membrane phospholipids with ion channels provide energetic stability and proper functioning of these channels (Schmidt et al., 2006; Xu et al., 2008; Hite et al., 2014; Ahuja et al., 2015). Further work is needed to distinguish between these two models.

This study expands the spectrum of Nav1.9-linked abnormality in nociception and confirms the significant role of the Nav1.9 channel in pain signaling. We show that the N816K mutation is likely to alter the local structure of the activation gate, confers gain-of-function attributes on Nav1.9 channels, which lead to hyperexcitability in small DRG neurons, is consistent with the pain experience by our patient. Thus, development of Nav1.9 specific inhibitors holds promise for effective pain management in the future. Moreover, side-effects could be minimal due to the low homology in sequence between Nav1.9 and other voltage-gated sodium channel isoforms, as well as the preferential expression of Nav1.9 in nociceptors in peripheral nervous system.

## Data Availability

The datasets generated for this study are available on request to the corresponding author.

## Ethics Statement

Human Subject Research: The studies involving human participants were reviewed and approved by the Yale Human Research Protection Program Institutional Review Boards and by the local ethics committee at the Uniklinik under an approved institutional review board. Written informed consent to participate in this study was provided by the participants’ legal guardian/next of kin. Animal Subjects: The animal study was reviewed and approved by the VA-CT Institutional Animal Care Use Committee.

## Author Contributions

JH performed the electrophysiological recordings, analyzed the data, and prepared the graphs. ME performed the *in silico* modeling of Nav1.9 channel. PZ isolated and transfected the primary neurons. FD-H provided the critical reagents. BS consented and enrolled the family in this study. KB provided the clinical data. AA and IK identified the N816K mutation of hNav1.9 channel in this family. SD-H and SW designed and supervised the project. All authors contributed to the writing of the manuscript.

## Conflict of Interest Statement

The authors declare that the research was conducted in the absence of any commercial or financial relationships that could be construed as a potential conflict of interest.

## Footnotes

1 https://gnomad.broadinstitute.org/

## References

[B1] Ahuja S. Mukund S. Deng L. Khakh K. Chang E. Ho H. (2015). Structural basis of Nav1.7 inhibition by an isoform-selective small-molecule antagonist. *Science* 350:aac5464. 10.1126/science.aac5464 26680203

[B2] Amaya F. Wang H. Costigan M. Allchorne A. J. Hatcher J. P. Egerton J. (2006). The voltage-gated sodium channel Nav1.9 is an effector of peripheral inflammatory pain hypersensitivity. *J. Neurosci.* 26 12852–12860. 10.1523/jneurosci.4015-06.2006 17167076 PMC6674969

[B3] Atkins J. F. Wills N. M. Loughran G. Wu C. Y. Parsawar K. Ryan M. D. (2007). A case for “StopGo”: reprogramming translation to augment codon meaning of GGN by promoting unconventional termination (Stop) after addition of glycine and then allowing continued translation (Go). *RNA* 13 803–810. 10.1261/rna.487907 17456564 PMC1869043

[B4] Bennett D. L. Clark A. J. Huang J. Waxman S. G. Dib-Hajj S. D. (2019). The role of voltage-gated sodium channels in pain signaling. *Physiol. Rev.* 99 1079–1151. 10.1152/physrev.00052.2017 30672368

[B5] Black J. A. Waxman S. G. (2002). Molecular identities of two tetrodotoxin-resistant sodium channels in corneal axons. *Exp. Eye Res.* 75 193–199. 10.1006/exer.2002.2014 12137764

[B6] Choi J. S. Cheng X. Foster E. Leffler A. Tyrrell L. Te Morsche R. H. (2010). Alternative splicing may contribute to time-dependent manifestation of inherited erythromelalgia. *Brain* 133 1823–1835. 10.1093/brain/awq114 20478850

[B7] Coste B. Osorio N. Padilla F. Crest M. Delmas P. (2004). Gating and modulation of presumptive NaV1.9 channels in enteric and spinal sensory neurons. *Mol. Cell Neurosci.* 26 123–134. 10.1016/j.mcn.2004.01.015 15121184

[B8] Cummins T. R. Dib-Hajj S. D. Black J. A. Akopian A. N. Wood J. N. Waxman S. G. (1999). A novel persistent tetrodotoxin-resistant sodium current In SNS-null and wild-type small primary sensory neurons. *J. Neurosci.* 19:RC43. 10594087 10.1523/JNEUROSCI.19-24-j0001.1999 PMC6784927

[B9] Dib-Hajj S. D. Choi J. S. Macala L. J. Tyrrell L. Black J. A. Cummins T. R. (2009). Transfection of rat or mouse neurons by biolistics or electroporation. *Nat. Protoc.* 4 1118–1126. 10.1038/nprot.2009.90 19617884

[B10] Dib-Hajj S. D. Rush A. M. Cummins T. R. Hisama F. M. Novella S. Tyrrell L. (2005). Gain-of-function mutation in Nav1.7 in familial erythromelalgia induces bursting of sensory neurons. *Brain* 128 1847–1854. 10.1093/brain/awh514 15958509

[B11] Dib-Hajj S. D. Tyrrell L. Black J. A. Waxman S. G. (1998). NaN, a novel voltage-gated Na channel, is expressed preferentially in peripheral sensory neurons and down-regulated after axotomy. *Proc. Natl. Acad. Sci. U.S.A.* 95 8963–8968. 10.1073/pnas.95.15.8963 9671787 PMC21185

[B12] Dib-Hajj S. D. Tyrrell L. Cummins T. R. Black J. A. Wood P. M. Waxman S. G. (1999). Two tetrodotoxin-resistant sodium channels in human dorsal root ganglion neurons. *FEBS Lett.* 462 117–120. 10.1016/s0014-5793(99)01519-7 10580103

[B13] Eberhardt E. Havlicek S. Schmidt D. Link A. S. Neacsu C. Kohl Z. (2015). Pattern of functional TTX-Resistant sodium channels reveals a developmental stage of human iPSC- and ESC-Derived nociceptors. *Stem Cell Rep.* 5 305–313. 10.1016/j.stemcr.2015.07.010 26321143 PMC4618592

[B14] Estacion M. Han C. Choi J. S. Hoeijmakers J. G. Lauria G. Drenth J. P. (2011). Intra- and interfamily phenotypic diversity in pain syndromes associated with a gain-of-function variant of NaV1.7. *Mol. Pain* 7:92. 10.1186/1744-8069-7-92 22136189 PMC3248882

[B15] Fjell J. Hjelmstrom P. Hormuzdiar W. Milenkovic M. Aglieco F. Tyrrell L. (2000). Localization of the tetrodotoxin-resistant sodium channel NaN in nociceptors. *Neuroreport* 11 199–202. 10.1097/00001756-200001170-00039 10683857

[B16] Han C. Yang Y. De Greef B. T. Hoeijmakers J. G. Gerrits M. M. Verhamme C. (2015). The Domain II S4-S5 Linker in Nav1.9: a missense mutation enhances activation, impairs fast inactivation, and produces human painful neuropathy. *Neuromolecular Med.* 17 158–169. 10.1007/s12017-015-8347-9 25791876

[B17] Han C. Yang Y. Te Morsche R. H. Drenth J. P. Politei J. M. Waxman S. G. (2017). Familial gain-of-function Nav1.9 mutation in a painful channelopathy. *J. Neurol. Neurosurg. Psychiatry* 88 233–240. 10.1136/jnnp-2016-313804 27503742

[B18] Hite R. K. Butterwick J. A. Mackinnon R. (2014). Phosphatidic acid modulation of Kv channel voltage sensor function. *eLife* 3:e04366. 10.7554/eLife.04366 25285449 PMC4212207

[B19] Hockley J. R. Boundouki G. Cibert-Goton V. Mcguire C. Yip P. K. Chan C. (2014). Multiple roles for Na1.9 in the activation of visceral afferents by noxious inflammatory, mechanical, and human disease-derived stimuli. *Pain* 155 1962–1975. 10.1016/j.pain.2014.06.015 24972070 PMC4220011

[B20] Huang J. Han C. Estacion M. Vasylyev D. Hoeijmakers J. G. Gerrits M. M. (2014). Gain-of-function mutations in sodium channel Nav1.9 in painful neuropathy. *Brain* 137 1627–1642. 10.1093/brain/awu079 24776970

[B21] Huang J. Vanoye C. G. Cutts A. Goldberg Y. P. Dib-Hajj S. D. Cohen C. J. (2017). Sodium channel NaV1.9 mutations associated with insensitivity to pain dampen neuronal excitability. *J. Clin. Invest.* 127 2805–2814. 10.1172/JCI92373 28530638 PMC5490760

[B22] King M. K. Leipold E. Goehringer J. M. Kurth I. Challman T. D. (2017). Pain insensitivity: distal S6-segment mutations in NaV1.9 emerge as critical hotspot. *Neurogenetics* 18 179–181. 10.1007/s10048-017-0513-9 28289907

[B23] Lampert A. O’reilly A. O. Dib-Hajj S. D. Tyrrell L. Wallace B. A. Waxman S. G. (2008). A pore-blocking hydrophobic motif at the cytoplasmic aperture of the closed-state Nav1.7 channel is disrupted by the erythromelalgia-associated F1449V mutation. *J. Biol. Chem.* 283 24118–24127. 10.1074/jbc.M802900200 18550534 PMC3259749

[B24] Leipold E. Hanson-Kahn A. Frick M. Gong P. Bernstein J. A. Voigt M. (2015). Cold-aggravated pain in humans caused by a hyperactive NaV1.9 channel mutant. *Nat. Commun.* 6:10049. 10.1038/ncomms10049 26645915 PMC4686659

[B25] Leipold E. Liebmann L. Korenke G. C. Heinrich T. Giesselmann S. Baets J. (2013). A de novo gain-of-function mutation in SCN11A causes loss of pain perception. *Nat. Genet.* 45 1399–1404. 10.1038/ng.2767 24036948

[B26] Lolignier S. Amsalem M. Maingret F. Padilla F. Gabriac M. Chapuy E. (2011). Nav1.9 channel contributes to mechanical and heat pain hypersensitivity induced by subacute and chronic inflammation. *PLoS One* 6:e23083. 10.1371/journal.pone.0023083 21857998 PMC3155549

[B27] Luke G. A. De Felipe P. Lukashev A. Kallioinen S. E. Bruno E. A. Ryan M. D. (2008). Occurrence, function and evolutionary origins of ‘2A-like’ sequences in virus genomes. *J. Gen. Virol.* 89 1036–1042. 10.1099/vir.0.83428-0 18343847 PMC2885027

[B28] Maingret F. Coste B. Padilla F. Clerc N. Crest M. Korogod S. M. (2008). Inflammatory mediators increase Nav1.9 current and excitability in nociceptors through a coincident detection mechanism. *J. Gen. Physiol.* 131 211–225. 10.1085/jgp.200709935 18270172 PMC2248717

[B29] Martin C. Stoffer C. Mohammadi M. Hugo J. Leipold E. Oehler B. (2018). NaV1.9 Potentiates Oxidized Phospholipid-Induced TRP responses only under inflammatory conditions. *Front. Mol. Neurosci.* 11:7. 10.3389/fnmol.2018.00007 29410612 PMC5787077

[B30] Mis M. A. Yang Y. Tanaka B. S. Gomis-Perez C. Liu S. Dib-Hajj F. (2019). Resilience to pain: a peripheral component identified using induced pluripotent stem cells and dynamic clamp. *J. Neurosci.* 39 382–392. 10.1523/JNEUROSCI.2433-18.2018 30459225 PMC6335750

[B31] O’Brien B. J. Caldwell J. H. Ehring G. R. Bumsted O’brien K. M. Luo S. Levinson S. R. (2008). Tetrodotoxin-resistant voltage-gated sodium channels Na(v)1.8 and Na(v)1.9 are expressed in the retina. *J. Comp. Neurol.* 508 940–951. 10.1002/cne.21701 18399542

[B32] O’Donnell A. M. Coyle D. Puri P. (2016). Decreased Nav1.9 channel expression in Hirschsprung’s disease. *J. Pediatr. Surg.* 51 1458–1461. 10.1016/j.jpedsurg.2016.05.007 27297039

[B33] Okuda H. Noguchi A. Kobayashi H. Kondo D. Harada K. H. Youssefian S. (2016). Infantile pain episodes associated with novel Nav1.9 mutations in familial episodic pain syndrome in japanese families. *PLoS One* 11:e0154827. 10.1371/journal.pone.0154827 27224030 PMC4880298

[B34] Osorio N. Korogod S. Delmas P. (2014). Specialized functions of nav1.5 and nav1.9 channels in electrogenesis of myenteric neurons in intact mouse Ganglia. *J. Neurosci.* 34 5233–5244. 10.1523/JNEUROSCI.0057-14.2014 24719102 PMC6609004

[B35] Padilla F. Couble M. L. Coste B. Maingret F. Clerc N. Crest M. (2007). Expression and localization of the Nav1.9 sodium channel in enteric neurons and in trigeminal sensory endings: implication for intestinal reflex function and orofacial pain. *Mol. Cell Neurosci.* 35 138–152. 10.1016/j.mcn.2007.02.008 17363266

[B36] Ritter A. M. Martin W. J. Thorneloe K. S. (2009). The voltage-gated sodium channel Nav1.9 is required for inflammation-based urinary bladder dysfunction. *Neurosci. Lett.* 452 28–32. 10.1016/j.neulet.2008.12.051 19146922

[B37] Rugiero F. Mistry M. Sage D. Black J. A. Waxman S. G. Crest M. (2003). Selective expression of a persistent tetrodotoxin-resistant Na+ current and NaV1.9 subunit in myenteric sensory neurons. *J. Neurosci.* 23 2715–2725. 10.1523/jneurosci.23-07-02715.2003 12684457 PMC6742082

[B38] Rush A. M. Waxman S. G. (2004). PGE2 increases the tetrodotoxin-resistant Nav1.9 sodium current in mouse DRG neurons via G-proteins. *Brain Res.* 1023 264–271. 10.1016/j.brainres.2004.07.042 15374752

[B39] Ryan M. D. Drew J. (1994). Foot-and-mouth disease virus 2A oligopeptide mediated cleavage of an artificial polyprotein. *EMBO J.* 13 928–933. 10.1002/j.1460-2075.1994.tb06337.x 8112307 PMC394894

[B40] Schmidt D. Jiang Q. X. Mackinnon R. (2006). Phospholipids and the origin of cationic gating charges in voltage sensors. *Nature* 444 775–779. 10.1038/nature05416 17136096

[B41] Subramanian N. Wetzel A. Dombert B. Yadav P. Havlicek S. Jablonka S. (2012). Role of Na(v)1.9 in activity-dependent axon growth in motoneurons. *Hum. Mol. Genet.* 21 3655–3667. 10.1093/hmg/dds195 22641814

[B42] Waxman S. G. Merkies I. S. Gerrits M. M. Dib-Hajj S. D. Lauria G. Cox J. J. (2014). Sodium channel genes in pain-related disorders: phenotype-genotype associations and recommendations for clinical use. *Lancet Neurol.* 13 1152–1160. 10.1016/S1474-4422(14)70150-4 25316021

[B43] Woods C. G. Babiker M. O. Horrocks I. Tolmie J. Kurth I. (2015). The phenotype of congenital insensitivity to pain due to the NaV1.9 variant p.L811P. *Eur. J. Hum. Genet.* 23 561–563. 10.1038/ejhg.2014.166 25118027 PMC4402639

[B44] Xu Y. Ramu Y. Lu Z. (2008). Removal of phospho-head groups of membrane lipids immobilizes voltage sensors of K+ channels. *Nature* 451 826–829. 10.1038/nature06618 18273018 PMC4026191

[B45] Zhang X. Y. Wen J. Yang W. Wang C. Gao L. Zheng L. H. (2013). Gain-of-Function mutations in SCN11A cause familial episodic pain. *Am. J. Hum. Genet.* 93 957–966. 10.1016/j.ajhg.2013.09.016 24207120 PMC3824123

